# Supplementary material for: Comprehensive somatic mutational analysis in glioblastoma: Implications for precision medicine approaches
Source: PLoS One. 2024 Jan 2;19(1):e0295698. doi: 10.1371/journal.pone.0295698 (PMC10760858; doi:10.1371/journal.pone.0295698)

**Supporting information**

**Contains all data for Supplementary Table 1-3 and Supplementary Figures 1-2.**

Supplementary Table 1:

Supplementary Table 2.


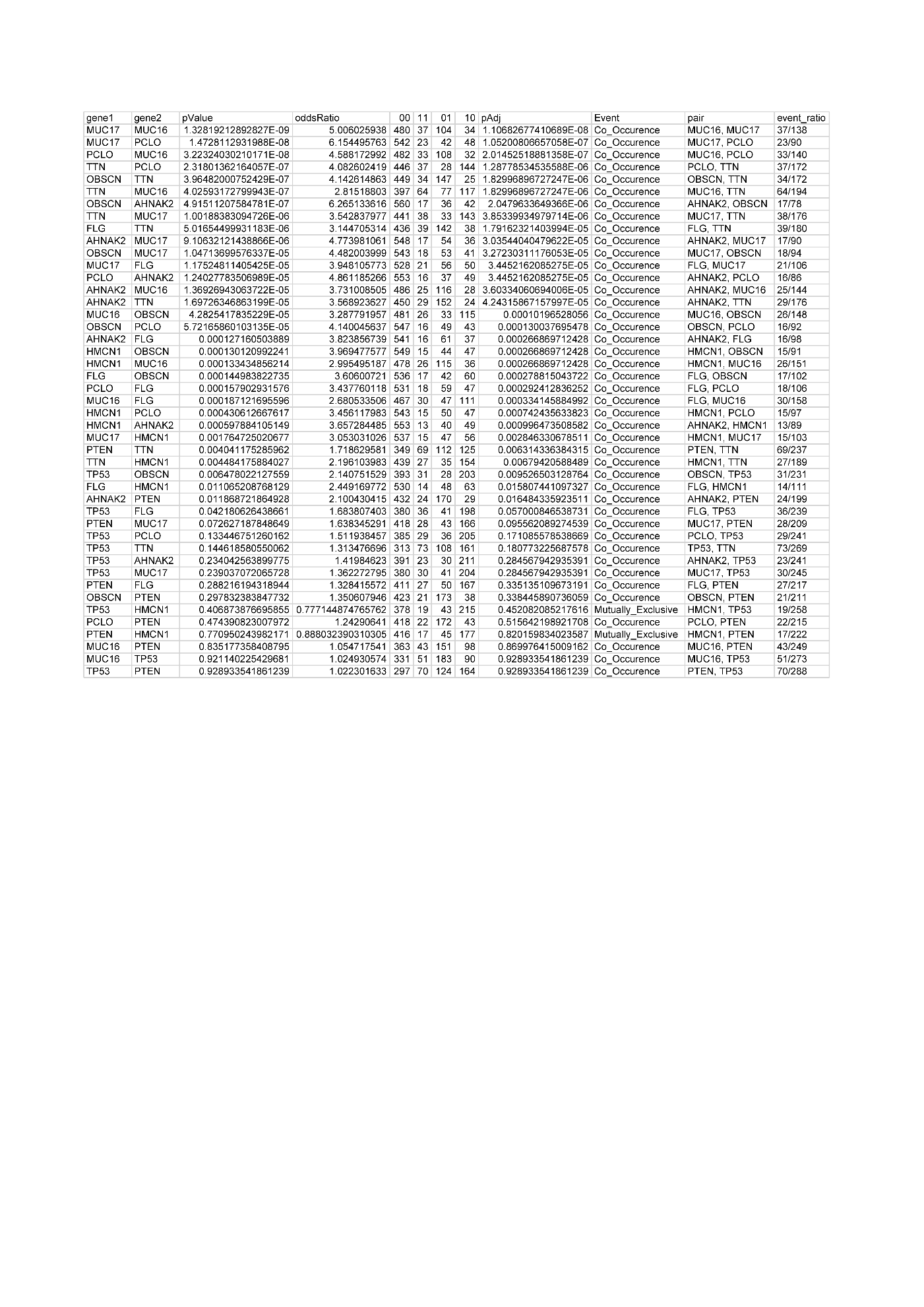


Supplementary Table 3.


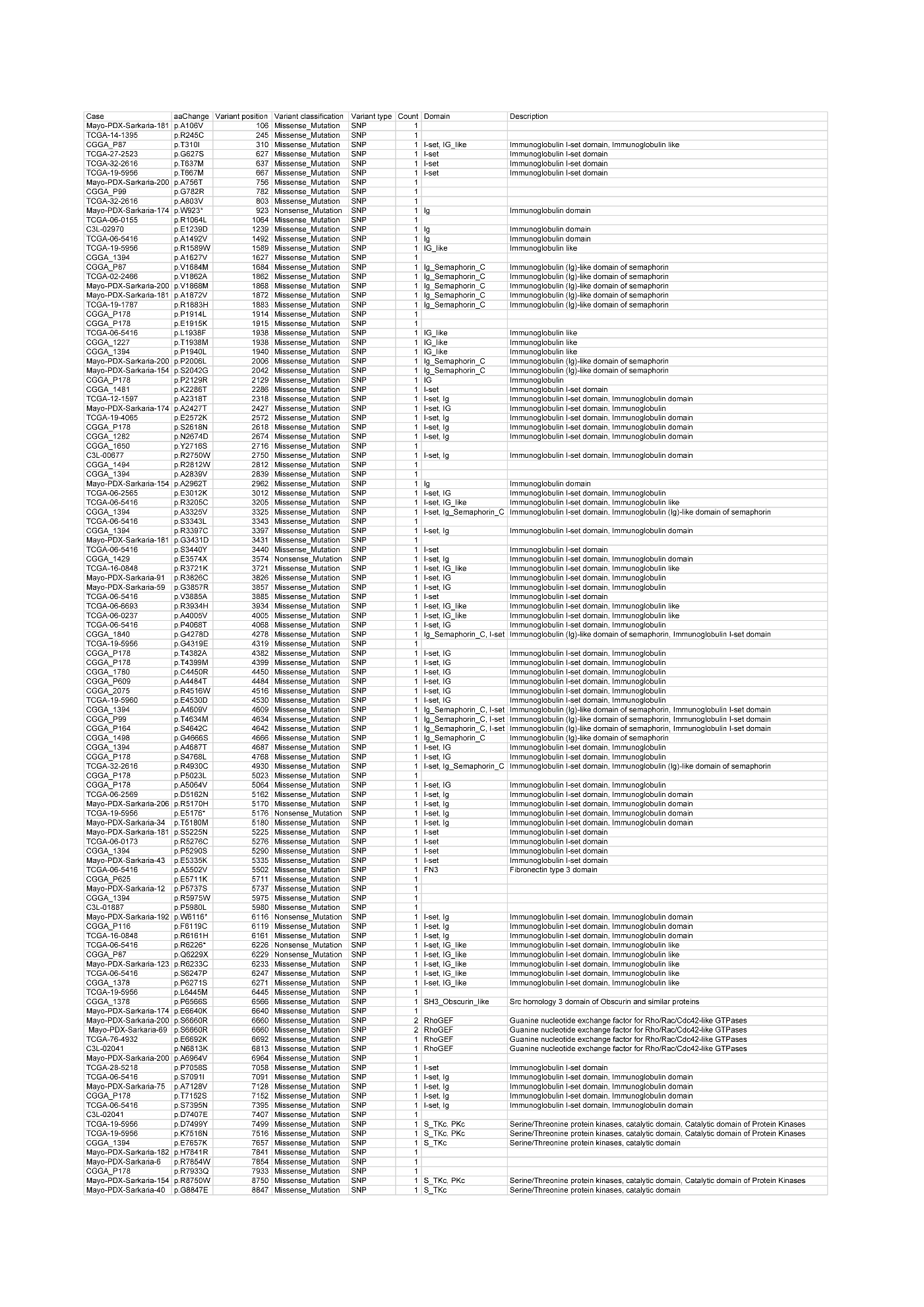


**Supplementary Figure 1.** Kaplan-Meier curves to analyze OS in GBM patients using an integrated CGGA, TCGA, CPTAC, and MAYO-PDX datasets for *PTEN, TP53, TTN, MUC16, FLG, PCLO, MUC17,* and *HMCN1* genes, based on their mutational status.


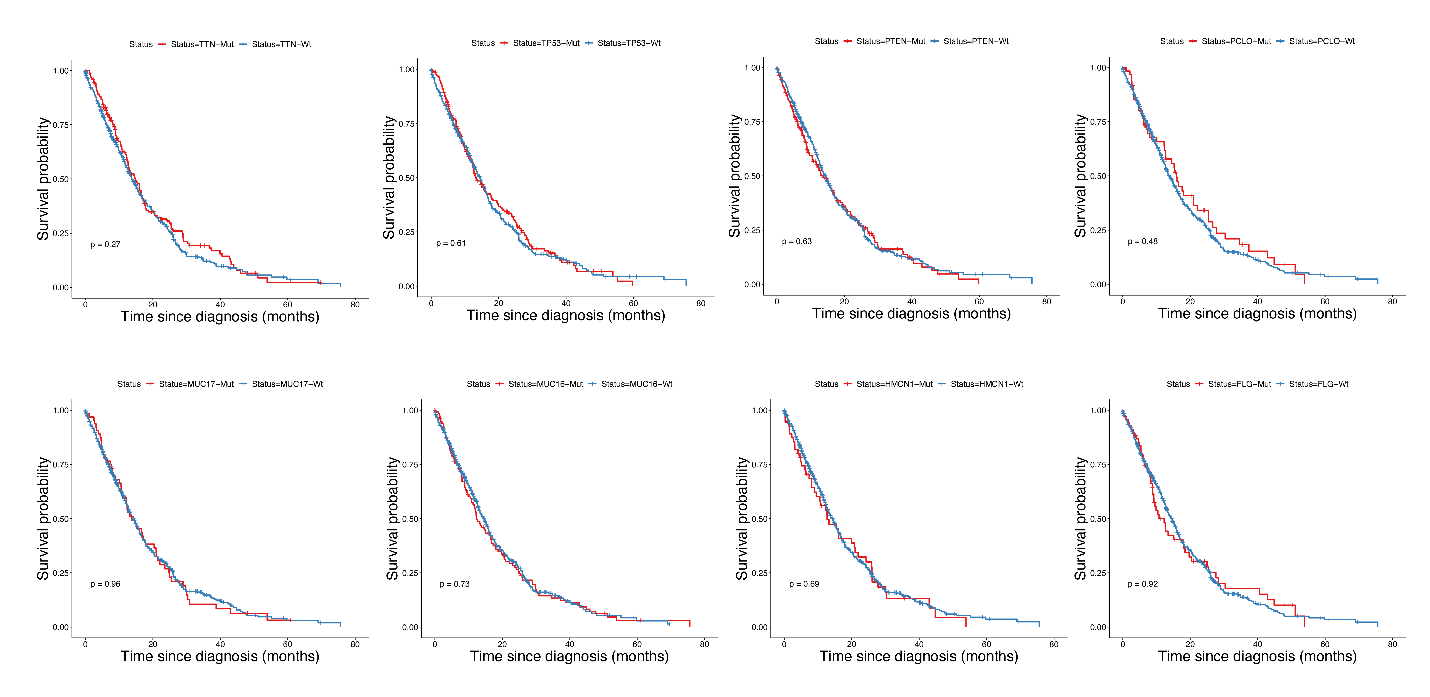


**Supplementary Figure 2.** Kaplan-Meier survival analysis for high and low expression levels of *OBSCN* and *AHNAK2* genes alone or in combination in CGGA, TCGA, CPTAC, and MAYO-PDX GBM datasets.


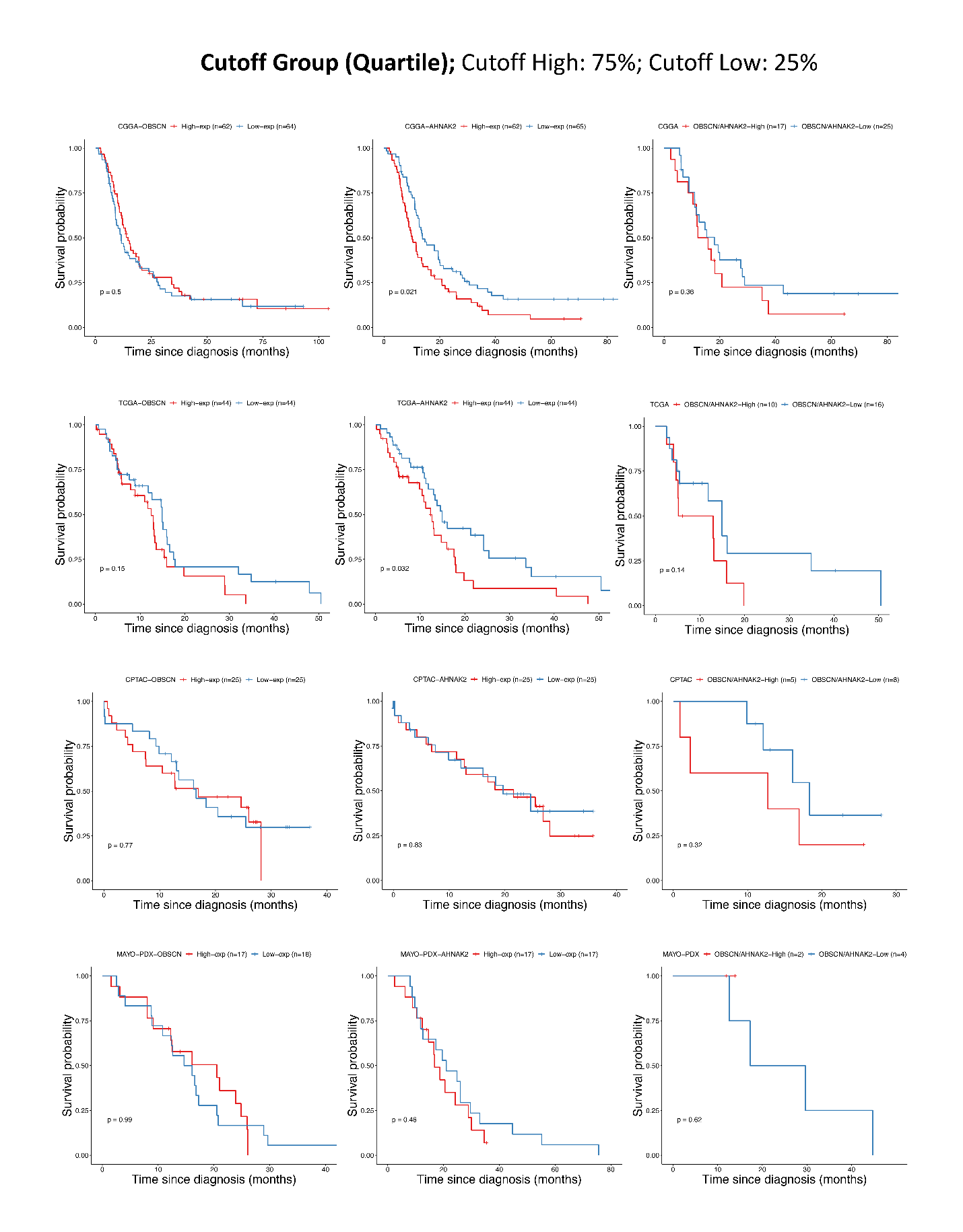

Supplement: S1 File — (DOCX) [file pone.0295698.s001.docx]
