# Supplementary material for: Comprehensive somatic mutational analysis in glioblastoma: Implications for precision medicine approaches
Source: PLoS One. 2024 Jan 2;19(1):e0295698. doi: 10.1371/journal.pone.0295698 (PMC10760858; doi:10.1371/journal.pone.0295698)
Supplement: Attachment — Submitted filename: Reviewers response.doc [file pone.0295698.s002.doc]

5 September 2023

**Dear Editorial Committee,**

*MS: No.*  PONE-D-23-20005

Comprehensive somatic mutational analysis in glioblastoma: implications for precision medicine approaches

Thank you for your e-mail. We found the reviewers’ comments very helpful. Please find the following point-by-point responses as requested:

**Comments to the Author**

1. Is the manuscript technically sound, and do the data support the conclusions?

The manuscript must describe a technically sound piece of scientific research with data that supports the conclusions. Experiments must have been conducted rigorously, with appropriate controls, replication, and sample sizes. The conclusions must be drawn appropriately based on the data presented.

Reviewer #1: Yes

Reviewer #2: Partly

2. Has the statistical analysis been performed appropriately and rigorously?

Reviewer #1: N/A

Reviewer #2: No

3. Have the authors made all data underlying the findings in their manuscript fully available?

The [PLOS Data policy](http://www.plosone.org/static/policies.action#sharing) requires authors to make all data underlying the findings described in their manuscript fully available without restriction, with rare exception (please refer to the Data Availability Statement in the manuscript PDF file). The data should be provided as part of the manuscript or its supporting information, or deposited to a public repository. For example, in addition to summary statistics, the data points behind means, medians and variance measures should be available. If there are restrictions on publicly sharing data—e.g. participant privacy or use of data from a third party—those must be specified.

Reviewer #1: Yes

Reviewer #2: Yes

4. Is the manuscript presented in an intelligible fashion and written in standard English? PLOS ONE does not copyedit accepted manuscripts, so the language in submitted articles must be clear, correct, and unambiguous. Any typographical or grammatical errors should be corrected at revision, so please note any specific errors here.

Reviewer #1: Yes

Reviewer #2: Yes

5. Review Comments to the Author

Please use the space provided to explain your answers to the questions above. You may also include additional comments for the author, including concerns about dual publication, research ethics, or publication ethics. (Please upload your review as an attachment if it exceeds 20,000 characters)

**Reviewer #1:**

This topic is very interesting, but some points must be revised. Look carefully at these:

- "We identified at least ten commonly mutated genes... associated with favorable survival outcomes in these patients." This part seems methods.

The following sentence “We identified at least ten commonly mutated genes in the investigated datasets including OBSCN and AHNAK2 genes. Upon integrating the mutational and clinical information, we discovered the co-mutation of OBSCN and AHNAK2 holds promise as a biomarker associated with favorable survival outcomes in these patients” was changed to “Our analysis revealed several commonly mutated genes, which could potentially indicate survival outcomes in GBM patients” in introduction section of new version as suggested.

- "Therefore, it is essential to identify new molecular targets that are involved in cell growth and survival to develop more effective therapeutic approaches for the GBM [4]" - In addition it is important to add the concept of "survivorship bias in glioblastoma". Consider PMID: 37392899.

Thank you for your comment. We have included the concept of "survivorship bias in glioblastoma" in the introduction section of the new version. “In addition, the development of prognostic GBM biomarkers often relies on surgically obtained tumor samples. However, bias due to varying surgeon selection criteria affects the accuracy of future analyses [12]. Thus, by integrating multiple datasets and considering larger sample sizes, it appears that the current limitations could potentially be mitigated.”

- "Notably, MUC17-MUC16, MUC17-PCLO, PCLO-MUC16, TTN-PCLO, and OBSCN-TTN interactions showed the strongest correlations" Are there other correlations between other genetic alterations?

Thank you for your inquiry. Apart from the mentioned correlations, we have provided additional significant interactions in new version of the Supplementary Table 2 for your reference.

- "While our study provides valuable insights into the potential. Some limitations should be taken into account". Among limitations authors should add that not only genetic mutations affect OS, but they also discuss the role of the tumor microenvironment (TME) and immune cell. Consider both these recent papers: -- PMID: 37218976 -- PMID: 34471502

Thank you for your valuable comments, we have added the suggested limitation into our discussion section. “Furthermore, the impact of the tumor microenvironment and immune cells, particularly macrophages, on glioma progression and survival has not been considered in our analysis [28-29]”

- "Our findings provide new insights into the molecular... for the development of molecular-targeted therapies." At the end of the manuscript, it is not clear whether this article is a review or clinical research. If this is clinical research, what do the authors add new compared to previous literature? Improve conclusion, what do they want to report new?

We have re-written the conclusion section of the new version file to emphasize the novel contributions of our study. The revised conclusion section is now more focused into GBM's molecular landscape and its relevance for developing targeted therapies. We believe these clarifications underscore the novelty and significance of our research.

The conclusion section was revised completely, as suggested by reviewers

**“Conclusion:** In summary, the integration of multiple GBM datasets has revealed critical mutations in the GBM landscape. Our study introduces a novel classification based on the mutation status of OBSCN and AHNAK2 among GBM patients. The distinct prognostic implications and molecular characteristics observed within the three OBSCN and AHNAK2 mutant phenotypes provide valuable insights. Notably, the Double-Mut phenotype has led to the identification of potential antitumor drugs, showing promise for customized therapies precisely designed for these specific mutant phenotypes. Moreover, our exploration of protein-level mutations within OBSCN and AHNAK2 has revealed the potential impact of specific protein domains on patient survival, suggesting that particular mutations within these domains could result in unique prognostic outcomes. Our study underscores the importance of precision medicine approaches in GBM treatment and highlights the potential of genetic marker analysis in improving patient outcomes. However, further investigation is needed to validate these findings and explore the therapeutic potential of OBSCN and AHNAK2 as potential biomarkers and targets for GBM treatment.”

**Reviewer #2:**

I appreciate the opportunity to review "Comprehensive somatic mutational analysis in glioblastoma: implications for precision medicine approaches" by Azimi et al. focusing on glioblastoma

multiforme (GBM), a topic of significant importance in oncology. The authors have chosen a rigorous approach and clear presentation of the material.

However, following careful consideration and review, I regretfully cannot recommend acceptance of this paper in its current form. My main concern is related to the novelty and impact of the findings. According to the data and methods employed in the manuscript, it seems that the work represents a reiteration of previous findings. The literature contains several published works that have produced almost identical results, utilizing similar methodologies. Moreover, the analysis employed, though robust in its design, could have been more comprehensive. Specifically, a systematic analysis including data from all available databases could have added more depth and relevance to the research. Instead, the study focused on a rather limited selection of the ten most common genes found in the specific datasets. This choice potentially limits the generalizability of the findings and may not fully represent the complexity of GBM.

In this study, we analyzed all available whole exome sequencing (WES) databases containing mutations of GBM patients, which include TCGA, CGGA, CPTAC and MAYO-PDX. The aim of this study was to find important genes that are mutated at a high rate in GBM patients using an unbiased bioinformatic data analysis. By our comprehensive bioinformatic data analysis, we have candidate a list of ten frequently mutated genes (PTEN, TP53, TTN, MUC16, FLG, PCLO, MUC17, HMCN1, AHNAK2, and OBSCN). Form our list, we found several novel candidate genes that is important to be discussed and considered in future. Among these ten genes, we selected those that had a significant relationship with overall survival and that had not been previously studies. However, we have prepared a supplementary table1 that shows all common mutated genes that highlighted by analyzing aforementioned four databases. In addition, we checked the overall survival for all these genes, which can be seen in the Supplementary Table 1.

Additionally, while the manuscript is well written and the data clearly presented, the conclusions drawn do not seem to be sufficiently supported by the data. For example, the identification of OBSCN and AHNAK2 mutations and their association with more favorable overall survival and specific drug sensitivity patterns seems very interesting; however, it is crucial that these observations are substantiated with more robust and specific data.

This section has re-written accordingly.

In summary, while the manuscript offers a worthwhile investigation of GBM, it would greatly benefit from further analyses to augment the novelty and impact of the findings. I believe that incorporating the above-mentioned considerations would significantly strengthen the manuscript, ultimately leading to a more rigorous and impactful contribution to the existing body of knowledge on GBM.

This was revised.

I hope that my feedback will be constructive for the authors and that the outlined concerns can be addressed in a revised version of the manuscript.

6. PLOS authors have the option to publish the peer review history of their article ([what does this mean?](https://journals.plos.org/plosone/s/editorial-and-peer-review-process#loc-peer-review-history)). If published, this will include your full peer review and any attached files.

If you choose “no”, your identity will remain anonymous but your review may still be made public.

**Do you want your identity to be public for this peer review?** For information about this choice, including consent withdrawal, please see our [Privacy Policy](https://www.plos.org/privacy-policy).

Reviewer #1: No

Reviewer #2: Yes: Georgios Markopoulos

I hope you find the corrections satisfactory.

Wish you all the best.

Yours sincerely

Azimi, P.
